# Anatomical study of a temporal bone from a non-human primate (*Callithrix sp*)

**DOI:** 10.1016/S1808-8694(15)30570-X

**Published:** 2015-10-19

**Authors:** Andrei Borin, Luciene Covolan, Luiz Eugênio Mello, Daniel Mochida Okada, Oswaldo Laércio Mendonça Cruz, Jose Ricardo Gurgel Testa

**Affiliations:** 1 Otorhinolaryngologist; MSc in Otorhinolaryngology, PhD student UNIFESP/EPM.; 2 PhD, Adjunct Professor, Department of Physiology - Federal University of São Paulo.; 3 MD, PhD, Full Professor. Department of Physiology - Federal University of São Paulo.; 4 MD. Otorhinolaryngologist.; 5 Associate Professor - Department of Otorhinolaryngology - Federal University of São Paulo. PhD. Adjunct Professor, Department of Otorhinolaryngology - Federal University of São Paulo. Federal University of São Paulo - Brasil.

**Keywords:** anatomy, animal, facial nerve, ear, temporal bone

## Abstract

The search for experimental (animal) models is essential to the development of clinical studies.

**Aim:**

To demonstrate, by means of micro dissection techniques, the anatomical structures of temporal bones from the primate *Callithrix sp*.

**Study design:**

Experimental.

**Methods:**

Dissection of temporal bone structures of *Callithrix sp* and photographic documentation.

**Results:**

We identified the main constituents of the temporal bone (external, medium and inner ear and facial nerve).

**Conclusion:**

The non-human primate *Callithrix sp*. is an adequate experimental model for the studies of temporal bone structures given its close anatomical similarities to that found in humans.

## INTRODUCTION

The investigation on new animal models in biological sciences represents a fundamental step in the search for scientific progresses that can be applied in day-to-day medicine. The study of physiology, physiopathology and the effects of new therapeutic approaches in experimental animals has become a mandatory precedent before carrying out clinical trials in humans. Otology also requires such progress, and many experimental models are reported in the literature. Small size mammals such as rats, mice and guinea pigs^1^, ^2^, ^3^, ^4^, ^5^ are commonly used in studies of temporal bones, and their anatomy and physiology are broadly described. Other mammals such as racoons^6^ and pigs^7^ are also well defined experimental models. However, the phylogenetic distance of such animals to human beings does not allow direct inference of the results obtained^8^. Moreover, factors such as body balance based on the quadruped position, a not very differentiated cochlear structure, a difficulty in assessing specific facial movements, amongst others, makes it even more difficult to study temporal bones in these animals. Anatomical-surgical studies in primates are being carried out in the literature to overcome these difficults^8^, ^9^, ^10^. The sagui used in the present investigation (Callithrix sp) is a native primate in Brazil, of small size, which is not under risk of extinction, and can reproduce in a cage and represents a low cost alternative to maintain studies in the many fields of knowledge. The saguis, like human beings, are primates, thus being animals very near each other in the phylogenetic scale.

Our proposal is to present a preliminary study on the temporal bone anatomy of the Callithrix sp with the goal of making feasible future efforts to define a new experimental model in otology.

## METHOD

We used four skulls (8 temporal bones) of species which were previously slaughtered as part of other studies on the central nervous system, previously approved by the Ethics in Research Committee of our Institution (Protocol # 1113/01). These animals were reared in our own institution. With the help of a surgical microscope (DF-Vasconcelos® M90) coupled to a digital camera (FUJI® F420), we then obtained corresponding images to the different stages of the dissection of such skulls, trying to prove the feasibility of carrying out the necessary surgical access in order to apply experimental paradigms to the temporal bone.

## RESULTS

**Figure 1:**
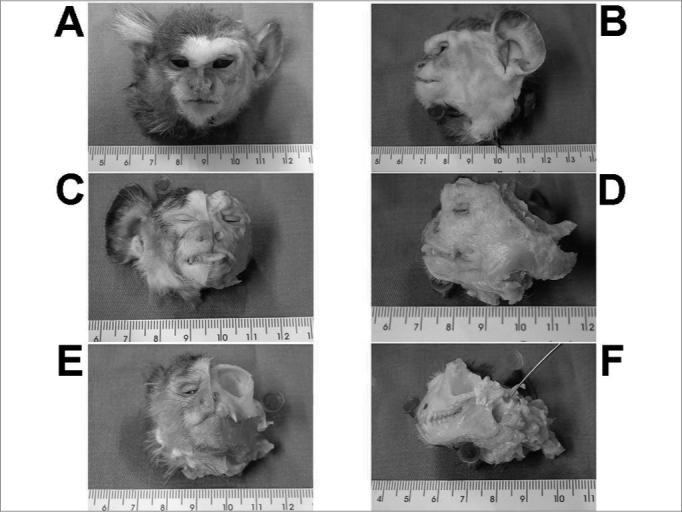
Macroscopic aspect of the skull and face. - Frontal (A, C, E) and lateral views (B, D, F) of the skull and face of animals with the skin (A, B), without the skin and adjacent structures (C, D) and without the muscles (E, F). Instrument tip showing the external acoustic meatus.

**Figure 2:**
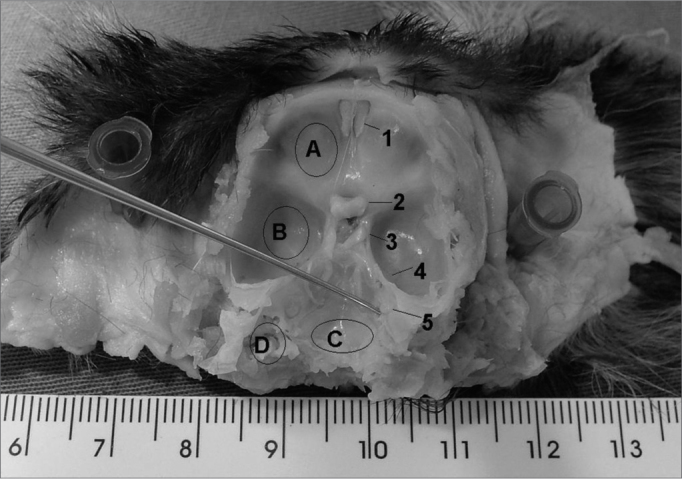
Upper view of the skull base. - A: anterior fossa; B: middle fossa; C: posterior fossa; D: mastoid; 1: olfactory nerve; 2: optic nerve; 3: oculomotor nerve; 4: trigeminal nerve ganglion; 5: internal acoustic meatus (instrument tip).

**Figure 3:**
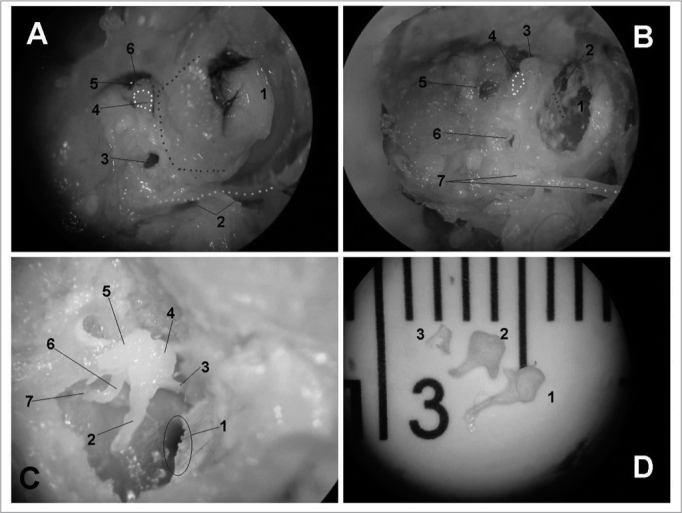
Middle ear dissection (right temporal bone). - View with intact external acoustic meatus - 1: External acoustic meatus; 2: extratemporal portion of the facial nerve; 3: posterior antrostomy; 4: incus; 5: malleus; 6: anterior antrostomy. B - partial removal of back wall - 1: tympanic membrane; 2: handle of malleus; 3: head of malleus; 4: short incus arm; 5: vestibule; 6: posterior antrostomy; 7: facial nerve. C - tympanic membrane and epitympanic wall removal - 1: Eustachian tube opening; 2: handle of malleus; 3: ear drum tensor muscle tendon; 4: head of malleus; 5: incus body; 6: incus long arm; 7: stapes. D - Ossicles - 1: malleus; 2: incus; 3: stapes (without footplate).

**Figure 4:**
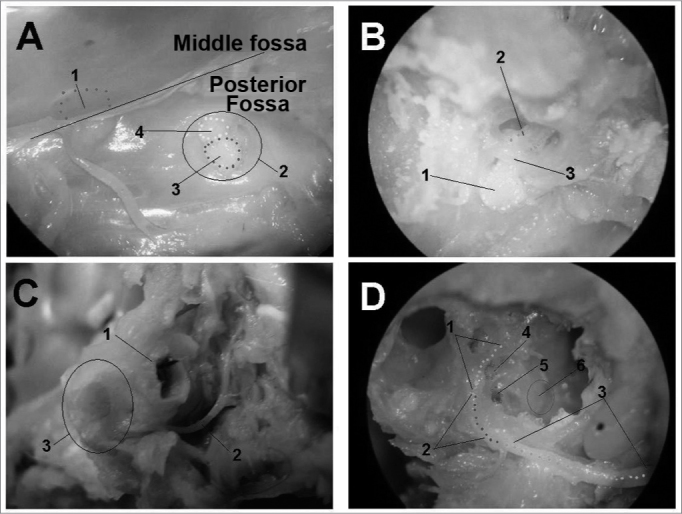
Facial nerve dissection (right temporal bone). - A - Upper view of the skull base - 1: Trigeminal ganglion; 2: internal acoustic meatus; 3: vestibule-cochlear nerve; 4: intracranial facial nerve. B - Internal acoustic meatus dissection - 1: intrameatal facial nerve; 2: labyrinthine facial nerve; 3: Fallopian canal entry. C - Extratemporal dissection - 1: External acoustic meatus; 2: extratemporal facial nerve; 3: mastoid. D - Middle ear medial wall - 1: Tympanic portion of the facial nerve; 2: mastoid portion of the facial nerve; 3: extratemporal portion of the facial nerve; 4: oval window; 5: round window; 6: promontory.

**Figure 5:**
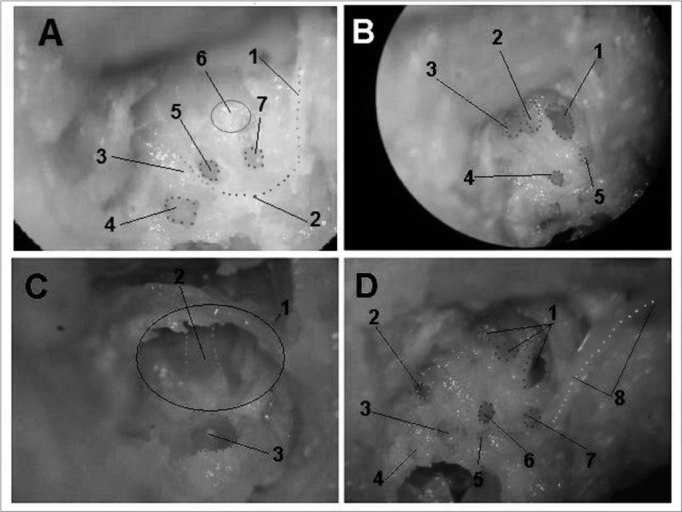
Inner ear dissection (right temporal bone) - A - 1: extratemporal portion of the facial nerve; 2: mastoid portion of the facial nerve; 3: Tympanic portion of the facial nerve; 4: vestibule; 5: oval window; 6: promontory; 7: round window. B - 1: cochlear basal turn; 2: cochlear middle turn; 3: cochlear apical turn; 4: oval window; 5: round window. C - 1: Eustachian Tube opening; 2: carotid (medial wall of the tube); 3: cochlear basal turn. D - 1: cochlea; 2: superior semi-circular canal opening; 3: vestibule; 4: posterior semi-circular canal opening; 5: lateral semi-circular canal opening; 6: oval window; 7: round window; 8: facial nerve.

Figure 1 depicts the macroscopic aspects of the skull and face of animals in dissections with and without the skin, connective tissue and muscles. Figure 2 represents an upper view of the skull base, showing the cranial nerves and the internal acoustic meatus. Figure 3 shows the middle ear dissection with visualization of the mastoid, anterior and posterior antrostomies, tympanic membrane and ossicles. Figure 4 shows the facial nerve in its intrameatal trajectory, labyrinthine, tympanic, mastoid and extratemporal. Figure 5 corresponds to the inner ear dissection, with the opening of the vestibules, the lateral canals, oval and round windows, promontory, cochlear turns, Eustachian Tube entry and carotid.

## DISCUSSION

Several experimental paradigms about the temporal bone have been proposed in different species of animals in the study of the ear (external, middle and internal) and the facial nerve (intra and extra-temporal). The Callithrix sp is promising in the application of these study models since surgically we have been able to locate the same anatomical structures mentioned in these papers^1^, ^2^, ^3^, ^4^, ^5^, ^6^, ^7^, ^8^, ^9^, ^10^. However, some details must be stressed.

Apparently, the facial nerve in these species has its extratemporal portion located under the parotid and not within it, as it happens to humans. This has been already described in rats^1^, and as we see it, facilitates its experimental handling. We did not see a stapedial artery that crosses over the round window niche as described in the rat5, absent in humans, and this would also represent an easiness to work in this region. Through posterior antrostomy we have access to the vestibule windows, where we could, for example, carry out gene transfer to the inner ear, as proposed in rats^5^. We see a cochlea made up of 2.5 turns, similar to that in humans (2.5-2.75 turns), and different from that of mice (1.5 turn) and guinea pigs (4.5 turns)6, and this means another advantage.

We have also noticed some difficulties. We were unable to open the internal acoustic meatus through the middle fossa without damaging the inner and middle ear structures, as it happens in humans. Apparently, the labyrinthine portion of the facial nerve is enclosed in the cochlear apical turn and the superior semi-circular canal, and this makes such access unfeasible. We were also unable to find a good access to the tympanic cavity through the external acoustic meatus by using ear specula, because of its reduced size and the tilt of its bony portion. This would make it very difficult to do transtympanic injection of substances.

## CONCLUSION

This preliminary microsurgical-anatomical study of the temporal bone of Callithrix sp suggests that this species can be eligible for otologic research in primates.
